# Adolescent binge ethanol impacts H3K36me3 regulation of synaptic genes

**DOI:** 10.3389/fnmol.2023.1082104

**Published:** 2023-03-03

**Authors:** Emily R. Brocato, Jennifer T. Wolstenholme

**Affiliations:** ^1^Department of Pharmacology and Toxicology, Virginia Commonwealth University, Richmond, VA, United States; ^2^VCU Alcohol Research Center, Virginia Commonwealth University, Richmond, VA, United States

**Keywords:** adolescent ethanol, alcohol, PFC, epigenetics, H3K36me3, RNA-seq, ChIP-seq, cryptic transcription

## Abstract

Adolescence is marked in part by the ongoing development of the prefrontal cortex (PFC). Binge ethanol use during this critical stage in neurodevelopment induces significant structural changes to the PFC, as well as cognitive and behavioral deficits that can last into adulthood. Previous studies showed that adolescent binge ethanol causes lasting deficits in working memory, decreases in the expression of chromatin remodeling genes responsible for the methylation of histone 3 lysine 36 (H3K36), and global decreases in H3K36 in the PFC. H3K36me3 is present within the coding region of actively-transcribed genes, and safeguards against aberrant, cryptic transcription by RNA Polymerase II. We hypothesize that altered methylation of H3K36 could play a role in adolescent binge ethanol-induced memory deficits. To investigate this at the molecular level, ethanol (4 g/kg, i.g.) or water was administered intermittently to adolescent mice. RNA-and ChIP-sequencing were then performed within the same tissue to determine gene expression changes and identify genes and loci where H3K36me3 was disrupted by ethanol. We further assessed ethanol-induced changes at the transcription level with differential exon-use and cryptic transcription analysis – a hallmark of decreased H3K36me3. Here, we found ethanol-induced changes to the gene expression and H3K36me3-regulation of synaptic-related genes in all our analyses. Notably, H3K36me3 was differentially trimethylated between ethanol and control conditions at synaptic-related genes, and *Snap25* and *Cplx1* showed evidence of cryptic transcription in males and females treated with ethanol during adolescence. Our results provide preliminary evidence that ethanol-induced changes to H3K36me3 during adolescent neurodevelopment may be linked to synaptic dysregulation at the transcriptional level, which may explain the reported ethanol-induced changes to PFC synaptic function.

## 1. Introduction

Adolescent development is characterized by a number of physiological, behavioral, emotional, and cognitive changes (Spear, 2000). These changes give rise to heightened reward sensitivity, sensation seeking, and impulsivity, and allow for increased participation in risky behaviors, such as the initiation and escalation of alcohol use (Spear, 2000; Casey et al., 2008; Romer et al., 2017; Lees et al., 2020). In a 2020 report, 16.1% of Americans between the age of 12–20 reported participation in alcohol consumption, with an estimated 9.2% participating in binge drinking (SAMHSA, 2020). Binge alcohol consumption, common during adolescence, is defined as having five or more drinks on one occasion (SAMHSA, 2020). Adolescents respond differently to alcohol than adults, showing increased sensitivity to alcohol’s rewarding aspects, and decreased sensitivity to alcohol’s aversive aspects (Spear, 2014). This altered sensitivity enables adolescents to consume more alcohol, giving rise to the binge drinking commonly seen among this age group, while subsequently increasing the risk for developing an alcohol use disorder (AUD) later in life (Grant and Dawson, 1997; Spear, 2000). Adolescent binge drinking is particularly harmful as the brain is still undergoing development throughout this period (Spear, 2000). Alcohol use can disrupt ongoing developmental processes, resulting in structural, cognitive, and behavioral effects (De Bellis et al., 2005; Medina et al., 2008; Bava et al., 2013; Squeglia et al., 2014; Lees et al., 2020; El Marroun et al., 2021), some of which last into adulthood (Pascual et al., 2007; Coleman et al., 2011, 2014; Vargas et al., 2014; Crews et al., 2016; Marco et al., 2017; Wolstenholme et al., 2017).

The prefrontal cortex (PFC) integrates information from several brain regions to regulate attention, executive function, and working memory (Spear, 2000). The PFC is one of the last brain regions to mature, and undergoes significant changes in adolescent neurodevelopment – evidenced by increased synaptic pruning and myelination (Gogtay et al., 2004; Barnea-Goraly et al., 2005; Drzewiecki et al., 2017). Human imaging studies show that adolescent alcohol use is associated with alterations in PFC structure and myelination (De Bellis et al., 2005; Medina et al., 2008; Pfefferbaum et al., 2018; El Marroun et al., 2021), and similar effects have been shown in rodents (Vargas et al., 2014; Montesinos et al., 2015; Vetreno et al., 2016; Wolstenholme et al., 2017; Tavares et al., 2019). This ethanol-induced damage to the PFC may play a role in the behavioral effects seen in response to adolescent binge ethanol, specifically in terms of memory function (Bekinschtein and Weisstaub, 2014; Vargas et al., 2014; Montesinos et al., 2015; Warburton and Brown, 2015; Marco et al., 2017; Wolstenholme et al., 2017; Macht et al., 2020; Bent et al., 2022; van Hees et al., 2022).

Binge ethanol use during adolescence can result in diminished cognitive function, including deficits in conditioned discrimination, reversal learning, and memory (Pascual et al., 2007, 2021; Coleman et al., 2011, 2014; Hanson et al., 2011; Mota et al., 2013; Vargas et al., 2014; Montesinos et al., 2015; Vetreno and Crews, 2015; Vetreno et al., 2016, 2020; Carbia et al., 2017; Marco et al., 2017; Wolstenholme et al., 2017; Contreras et al., 2019; Drissi et al., 2020; Macht et al., 2020; Peñasco et al., 2020; Bent et al., 2022; van Hees et al., 2022). In rodents, adolescent binge ethanol leads to long-term deficits in working memory (Vargas et al., 2014; Marco et al., 2017; Wolstenholme et al., 2017; Bent et al., 2022) and cognitive flexibility (Coleman et al., 2011; Vetreno and Crews, 2012; Acheson et al., 2013; Coleman et al., 2014; Gass et al., 2014). Moreover, it has been shown in rodent (Markwiese et al., 1998; White et al., 2000; Younis et al., 2019; Bent et al., 2022) and human (Acheson et al., 1998) studies that ethanol-induced behavioral effects are more substantial when ethanol exposure takes place during adolescence than in adulthood, suggesting that adolescence is a critical time for this damage to occur. Memory deficits in a novel object recognition (NOR) task occurred in adolescent mice exposed to binge ethanol (Wolstenholme et al., 2017; Bent et al., 2022), but did not occur when ethanol exposure occurred in adulthood (Bent et al., 2022). While the behavioral and cognitive effects of adolescent binge ethanol have been widely studied, the mechanisms behind how these behavioral effects occur is largely unknown.

Recent findings have implicated histone methylation as playing a critical role in regulating psychiatric disorders, including AUD (Tsankova et al., 2007; Robison and Nestler, 2012; Krishnan et al., 2014; Barbier et al., 2017; Berkel and Pandey, 2017; Pandey et al., 2017), and ethanol dysregulates histone methylation in both human (Ponomarev et al., 2012) and rodent models (Finegersh and Homanics, 2014; Montesinos et al., 2016; Barbier et al., 2017; Kyzar et al., 2017; Wolstenholme et al., 2017). The stable, yet highly dynamic nature of these histone methylation marks also makes them particularly suitable for involvement in learning and memory processes (Parkel et al., 2013). Given these data, ethanol exposure during the critical adolescent period could alter histone methylation in specific brain regions to affect behavioral outcomes into adulthood. While a recent study has utilized epigenomic editing to modulate adolescent ethanol-induced behavioral changes in rats (Bohnsack et al., 2022), the connection between ethanol-induced epigenetic changes in the brain and behavioral deficits requires additional investigation.

Previously, it was shown that adolescent binge ethanol leads to persistent memory deficits in a NOR task (Montesinos et al., 2015; Vetreno and Crews, 2015; Vetreno et al., 2016; Marco et al., 2017; Wolstenholme et al., 2017; Drissi et al., 2020; Macht et al., 2020; Peñasco et al., 2020; Pascual et al., 2021; Bent et al., 2022; van Hees et al., 2022), alterations in adult spatial memory (Contreras et al., 2019; Bent et al., 2022), dysregulation of histone methyltransferases (HMTs) specific for H3K36(Wolstenholme et al., 2017), and global decreases of H3K36 levels in the PFC (Wolstenholme et al., 2017). Dysregulation of H3K36 may underlie the memory deficits associated with adolescent binge ethanol, due to the role H3K36 methylation plays in supporting transcriptional fidelity and memory consolidation (Butler and Dent, 2012; Gräff et al., 2012). H3K36me3 is enriched within transcribed regions of genes, and acts as a safeguard to protect against aberrant transcription (Smolle et al., 2013). After RNA Polymerase II (RNA pol II) transcribes a sequence, chromatin is “reset” to maintain proper transcriptional fidelity (Smolle et al., 2013). H3K36me3 plays a key role in this, recruiting other factors to de-acetylate histones H3 and H4, and maintaining ordered chromatin over open reading frames to repress unwanted transcription from occurring (Carrozza et al., 2005; Smolle and Workman, 2013). When H3K36me3 is not present, chromatin remains open and becomes subject to what is considered “cryptic” or aberrant transcription – RNA pol II can begin transcription from the middle of the coding region of the gene, instead of beginning at the correct transcription start site, ultimately producing an incorrect, truncated transcript (Wei et al., 2019). These effects can be seen with knockdown of *Setd2*, the specific HMT for H3K36me3. In mouse embryonic stem cells, *Setd2* knockdown resulted in increased number of cryptic transcripts, hallmarks of which include transcription of infrequently transcribed genes, dysregulation of intragenic transcriptional initiation, and alternative splicing patterns (Zhang et al., 2014). A reduction in H3K36me3 in a mouse model also gave rise to impaired splicing, altered gene expression, and ultimately, cognitive deficits (Sessa et al., 2019). Cryptic transcripts have the potential to interfere with the full-length gene product, leading to gene expression discrepancies (Butler and Dent, 2012). H3K36 dysregulation has been associated with a number of neurological diseases, such as Autism Spectrum Disorder (Sessa et al., 2019), and H3K36me3-mediated alternative splicing was found to play a role in cocaine reward behavior (Xu et al., 2021). Given this data, we hypothesized that adolescent binge ethanol negatively affects trimethylation of H3K36 within the PFC, leading to transcriptional changes in genes impacting memory-related processes.

Using RNA-seq and chromatin immunoprecipitation coupled to sequencing (ChIP-seq), we determined which H3K36me3-regulated loci were differentially affected by adolescent binge ethanol, and how binge ethanol affects gene expression. Here, we found that adolescent binge ethanol impacted the regulation and expression of genes important for synaptic function and memory processes. Ultimately, this work identified novel genes that may contribute to the persistent memory deficits associated with binge ethanol.

## 2. Results

**Figure 1:**
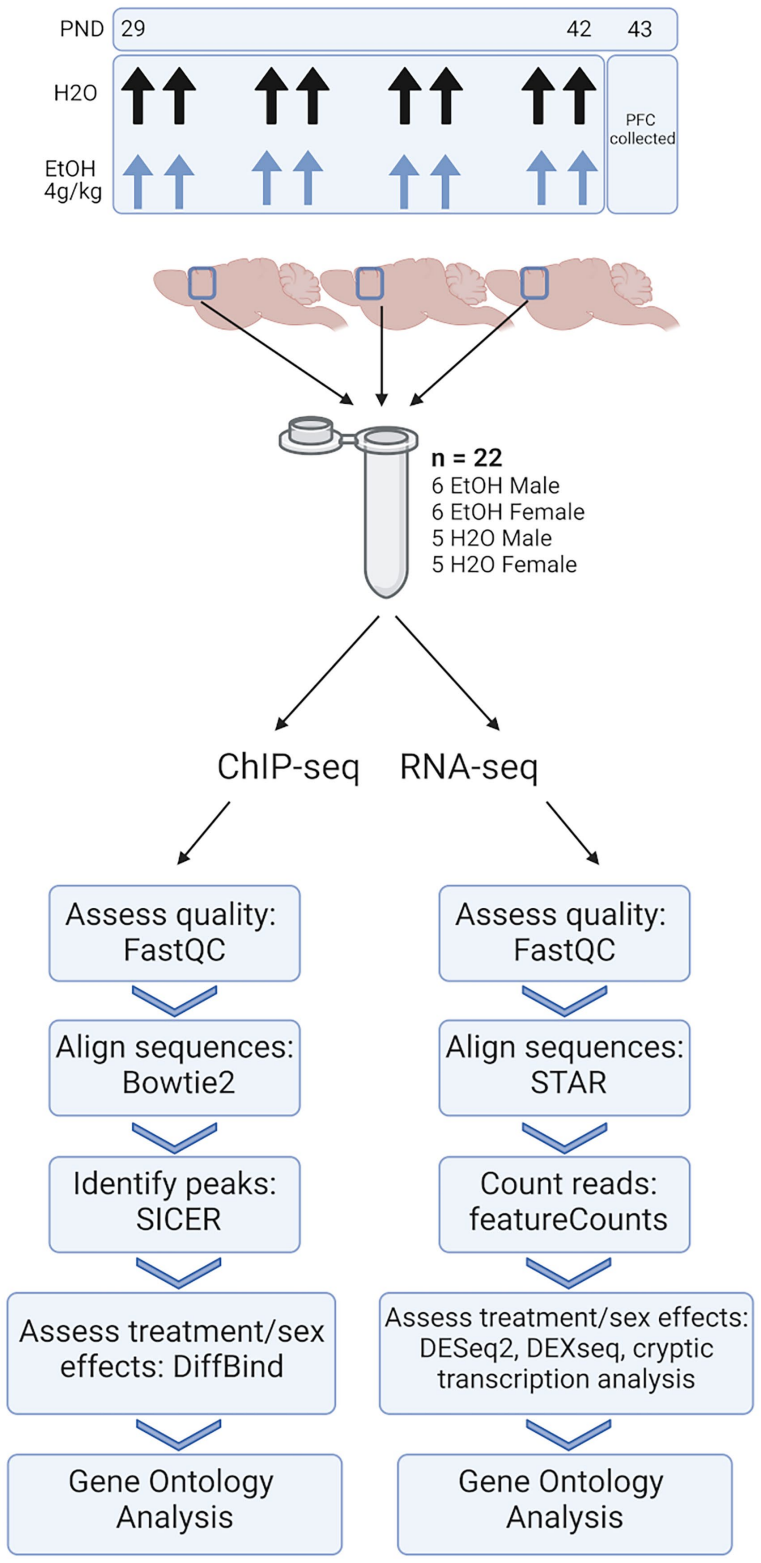
Methods. Adolescent DBA/2J mice were dosed with water or ethanol (4 g/kg) from PND 29–42. PFC was collected 24 h after the last dose of ethanol. Three PFCs from each treatment group were homogenized together, divided, and subjected to ChIP-seq and RNA-seq analysis.

We conducted molecular analyses on chromatin and gene expression responses to adolescent binge ethanol exposure in male and female DBA/2J mice at multiple levels of resolution, including whole transcript, exon-utilization, and cryptic transcription. These analyses were done within the same tissue to determine within-sample changes at both the chromatin regulation and gene expression levels (Figure 1). We found divergent responses between male and female animals with consistent, but fewer common responses that occurred across the sexes. We therefore describe our findings below to explicitly highlight these sex-specific responses. While both sexes showed commonality between gene ontology terms, the genes contained within these categories were sometimes different, suggesting the possibility that ethanol may act on common pathways, but through different genes or signaling cascades.

### 2.1. Adolescent binge ethanol induces changes to extracellular matrix and axon guidance-related genes in males

**Figure 2:**
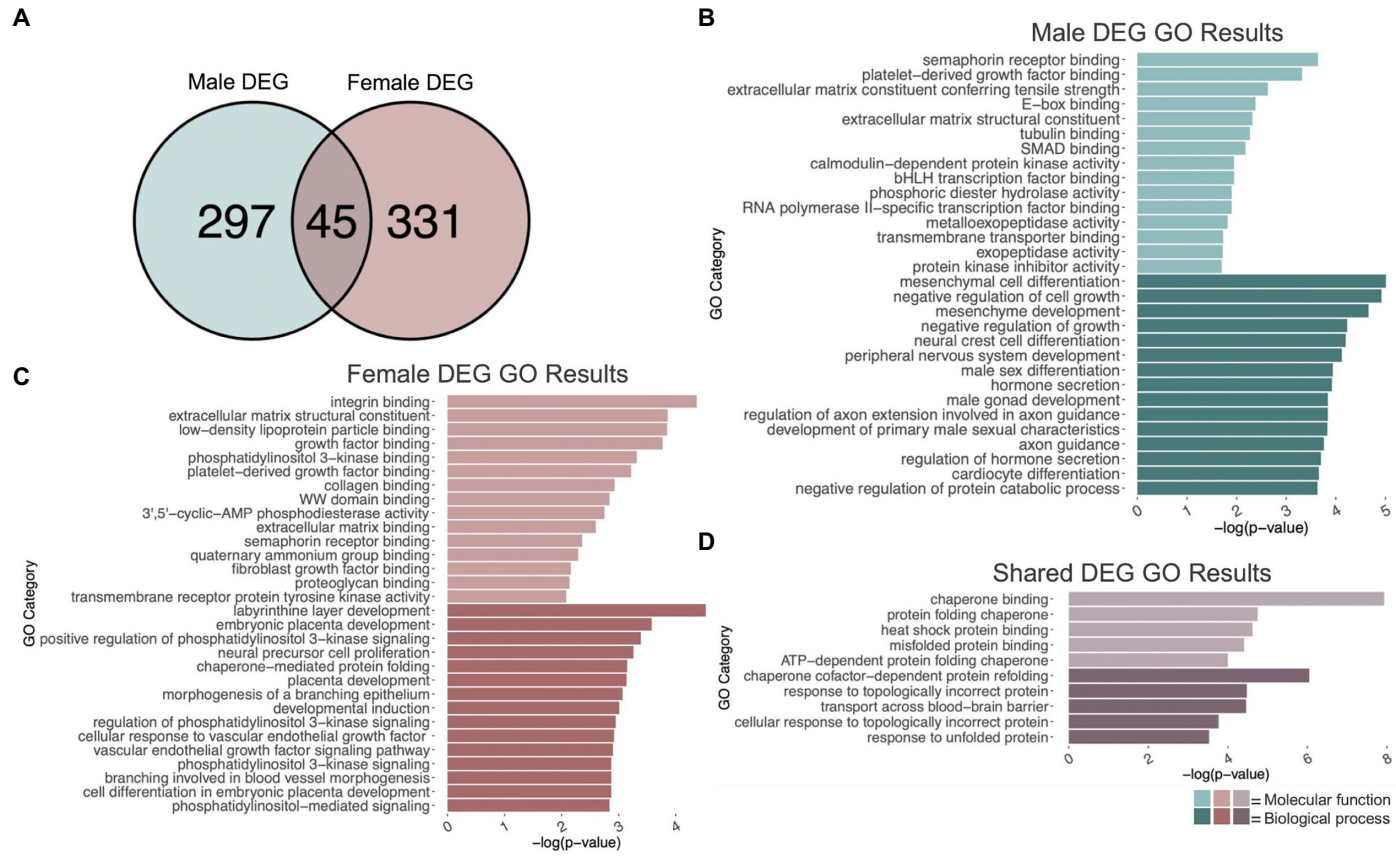
GO analysis of DEGs. **(A)** Number of genes differentially expressed due to adolescent binge ethanol in males and females at *p* < 0.01. Fisher’s exact test determined male and female gene overlap to be significant, *p* = 5.7e-34. **(B)** GO analysis of DEGs unique to males. **(C)** GO analysis of DEGs unique to females. **(D)** GO analysis of DEGs that are shared between males and females.

To identify genes that were differentially regulated by repeated binge ethanol in the PFC of DBA/2J male mice, we used DESeq2 with a value of *p* < 0.01 without additional filtering for fold-change to generate a gene list of sufficient length for gene discovery using downstream gene ontology analyses. Adolescent binge ethanol differentially regulated 342 total genes in males, with 175 being up-regulated and 167 down-regulated (Supplementary Table 1). Two hundred and ninety-seven of these genes were unique to males (Figure 2A) and served as the input for male-specific gene ontology over-representation analysis using ToppFun. Results of the gene ontology analysis are shown in Figure 2B. The first 15 molecular function and biological process categories were selected to represent our results. The full gene ontology results can be found in Supplementary Table 2. The top significant categories for molecular function included semaphorin binding, extracellular matrix structural constituent, tubulin binding, and calmodulin-dependent kinase activity. For biological process, top significant categories included negative regulation of cell growth, male sex differentiation, and axon guidance.

### 2.2. Adolescent binge ethanol induces changes to growth factor binding and extracellular matrix organization in females

Differential gene expression and gene ontology analysis were carried out for females as described above. At a value of *p* < 0.01, 376 genes were differentially expressed due to binge ethanol in females, with 215 up-regulated and 161 down-regulated (Supplementary Table 1). Three hundred and thirty-one genes were uniquely differentially regulated in female PFC (Figure 2A) and served as input for female-specific gene ontology analysis. Results of the female gene ontology analysis are shown in Figure 2C. Molecular function gene categories that were significantly altered by ethanol in females include extracellular matrix structural constituent, growth factor binding, and semaphorin receptor binding, similar categories to our male differential gene expression analysis. The top significant categories for biological process included positive regulation of phosphatidylinositol 3 kinase activity, neural precursor cell differentiation, and chaperone mediated protein folding. Although not included within the top 15 biological process categories, females also showed gene expression changes in genes involved in oligodendrocyte differentiation (*p* = 0.01, Supplementary Table 3). Reduced oligodendrocyte differentiation is thought to be an underlying cause of ethanol-induced myelin loss (Bichenkov and Ellingson, 2009; Kim et al., 2015; Darbinian et al., 2021; Guo et al., 2021), and has shown to be epigenetically regulated (Emery and Lu, 2015; Liu et al., 2015; Guo et al., 2021). Our lab has previously seen reduced myelin-related gene expression and reduced levels of H3K9me3, which was shown to regulate differentiation of oligodendrocyte precursor cells into mature oligodendrocytes (Liu et al., 2015; Wolstenholme et al., 2017).

While the extracellular matrix structural constituent category (GO:0005201) was found in both the male and female gene ontology analysis, the genes contained in each of these categories differed by sex. In males, *Ltbp4*, *Col4a1*, *Col4a2*, *Col9a3*, *Abi3bp*, *Tnc*, *Col6a6*, and *Agrn* in this category were differentially expressed, while in females, *Col24a1, Fbn1, Nid1, Ntn1, Col5a1, Hspg2, Creld2, Chadl, Thbs1, Igfbp7,* and *Eln* in this category were differentially expressed. Similarly, both male and female gene ontology analysis returned the semaphorin receptor binding category (GO:0030215) but with different genes represented. In males, *Sema3a, Sema5b, Sema4a,* and *Sema4b* were differentially expressed due to binge ethanol. In females, *Sh3bp1*, *Sema6a*, and *Sema3d* were differentially expressed. These results imply that the extracellular matrix and semaphorin binding is altered by adolescent binge ethanol in both males and females, but may be altered through a different mechanism in each sex.

### 2.3. Adolescent binge ethanol induces changes to unfolded protein response-related genes in males and females

Three hundred forty-two genes were differentially expressed in males, and 376 genes were differentially expressed in females due to adolescent binge ethanol at a value of *p* < 0.01. Of those, only 45 genes were differentially expressed in both males and females (*p* = 5.7e-34, Figure 2A). Due to the smaller number of genes for our shared gene ontology analysis, we selected the first five molecular function and biological process categories to represent our results (Figure 2D). Gene ontology analysis showed that these overlapping genes were related to chaperone binding (molecular function), misfolded protein binding (molecular function), chaperone-cofactor-dependent protein refolding (biological process), and transport across blood–brain barrier (biological process). The full gene ontology results can be found in Supplementary Table 4.

### 2.4. Adolescent binge ethanol induces differential exon use of dendritic spine-related genes in males and females

**Figure 3:**
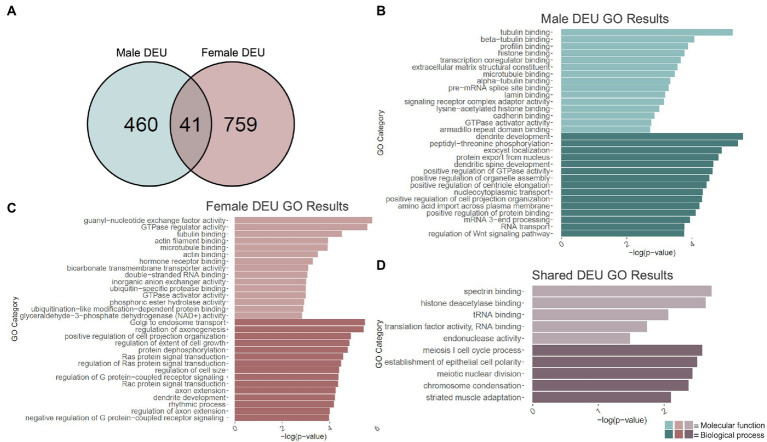
GO analysis of differential exon use (DEU) genes. **(A)** Number of genes showing differential exon use due to adolescent binge ethanol in males and females at *p* < 0.001. Fisher’s exact test determined male and female gene overlap to be significant, *p* = 1.7e-11. **(B)** GO analysis of genes with differentially used exons unique to males. **(C)** GO analysis of genes with differentially used exons unique to females. **(D)** GO analysis of genes with differentially used exons that are shared between males and females.

Given H3K36me3’s proposed role in alternative splicing (Zhou et al., 2014; Xu et al., 2021), we next performed an exon-level analysis of binge ethanol gene expression in males and females using DEXSeq. At a value of *p* < 0.001, males showed 595 unique differentially used exons within 501 genes due to binge ethanol treatment (Supplementary Table 5). Of those, 460 genes were unique to males, and were used as input for male-specific gene ontology analysis (Figure 3A). Significantly over-represented gene ontology categories for molecular function included tubulin binding, histone binding, and extracellular matrix structural constituent (Figure 3B). Top biological process categories included dendrite development, dendritic spine development, and positive regulation of GTPase activity (Figure 3B).

Exon-level analysis was carried out identically in females. At a value of *p* < 0.001, females showed 980 differentially used exons in 800 genes, almost double the number in males (Supplementary Table 5). Of those 759 genes were unique to females and were used as input into the female-specific gene ontology analysis (Figure 3A). Molecular function categories that were significantly over-represented included guanyl-nucleotide exchange factor activity, tubulin binding, actin filament binding, and microtubule binding (Figure 3C). Biological process categories that were over-represented included positive regulation of cell projection organization, axon extension, and dendrite development (Figure 3C).

DEXSeq analysis showed that after adolescent ethanol treatment, 501 genes had differentially used exons in males, and 800 genes showed differentially used exons in females at a value of *p* < 0.001. Of those, 41 genes showed differentially used exons in both males and females (*p* = 1.7e-11, Figure 3A). Significantly over-represented gene categories included histone deacetylase binding (molecular function), translation factor activity, RNA binding (molecular function), and chromatin condensation (biological process; Figure 3D). *Tnfrsf25* was the only gene in females that was differentially expressed due to binge ethanol, showed differential exon usage, and also showed evidence of cryptic transcription (Table 1). Full gene ontology results for males, females, and shared genes that showed differential exon usage can be found in Supplementary Tables 6–8.

**Table 1:** Genes that appeared in more than one analysis.

Gene name	DEG	DEU	CT	DBR	Gene name	DEG	DEU	CT	DBR	Gene name	DEG	DEU	CT	DBR	Gene name	DEG	DEU	CT	DBR
Atp11b					Tanc2					Gfpt1					Vsnl1				
Dnajc21					Trim24					Ide					Aqp4				
Lss					U2af2					Ino80					Mettl7a1				
Slc6a6					Vsig10					Jak1					Polr2b				
Penk					Wdr47					Klhdc4					Banp				
Ipo9					Zswim5					Mbip					Drc1				
Unc13a					Adcy1					Metap2					Klf3				
Hsph1					Arhgap32					Myo5a					Pde1a				
Smarca2					Arrdc3					Nalcn					Psmc2				
Tnfrsf25					Dlgap1					Nipal3					Tead4				
Atp13a5					Dmd					Phka1					Ubr3				
Cfap61					Efna5					Pik3cd					Aox1				
Crlf2					Elavl2					Pspc1					Ddx60				
Dnah6					Fut9					Rbm46					Gpt2				
Dtnb					Gbf1					Rft1					Hmgcr				
Herc1					Gramd1b					Rgs18					Ice1				
Htt					Gtf2ird1					Sidt1					Mphosph10				
Nek10					Itga6					Slc35d1					Pbk				
Nrxn3					Klhl2					Slc4a4					Rhobtb2				
Pclo					Mmp16					Ssbp3					Tnrc6c				
S100pbp					Ncam2					Tbc1d2b					Wdr43				
Tex2					Nemf					Thap4					Zfp949				
Vps8					Nrxn1					Thrap3					Hspa5				
Zic4					Phrf1					Thy1					Zbtb40				
Actr3b					Plcb1					Trrap					Adamts3				
Arhgef26					Ralyl					Vps13c					Fgfr1				
Cacnb1					Sos2					Zrsr2					Klc1				
Carm1					Srsf11					Itgb1					Mlycd				
Cnot1					Stoml1					Trabd2b					Oprk1				
Dock9					Vps13b					Arglu1					Tnik				
Farp1					Zmiz1					Dab2					Ap4s1				
Glyr1					Abhd2					Ltbp4					Snap25				
Gns					Btbd2					Psma6					Acot13				
Hdac9					Cacna2d3					Zkscan2					Plcl1				
Ica1					Cdc42bpb					Abi3bp					Pdia6				
Kctd1					Cdk13					Cited2					Usp27x				
Lrp1					Cep164					Col4a2					B9d1				
Lrrk2					Cers6					Cps1					Fkbp2				
Lsm8					Clasp2					Eif4a3					Per1				
Mtf2					Cpe					Kif5b					Dbp				
Picalm					Csmd2					Lbhd1					Rgs13				
Prrc2c					Dcx					Morf4l1					Bbln				
Ptpru					Elmo1					Nefh					Dclk3				
Rgs2					Esrrb					Nuak1					Triqk				
Ryr2					Etv4					Nudt16l1					Cplx1				
Sez6					Fam135b					Pde11a									
Sh2b1					Fam222b					Sash1							Unique to male analysis			
Slc17a5					Fubp1					Sema3a							Unique to female analysis			
Slc6a12					Gapvd1					Slc16a14							Found in male & female analyses				
Srgap2					Gemin6					Tmem101									

DEG, differentially expressed gene; DEU, differential exon use; CT, cryptic transcription; DBR, differentially bound region.

### 2.5. Adolescent binge ethanol induces cryptic transcription of *Snap25* and *Cplx1* in males and females

**Figure 4:**
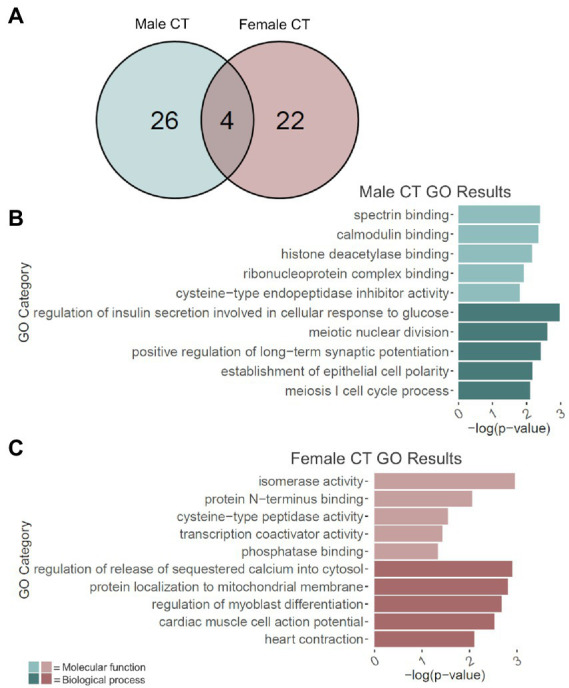
GO analysis of cryptically transcribed (CT) genes. **(A)** Number of genes cryptically transcribed due to adolescent binge ethanol in males and females at *p* < 0.05. Fisher’s exact test determined male and female gene overlap to be significant, *p* = 8.2e-09. **(B)** GO analysis of cryptically transcribed genes with unique to males. **(C)** GO analysis of cryptically transcribed genes unique to females. *Snap25*, *Cplx1*, *Ccdc124*, and *Per1* were cryptically transcribed in both males and females and were excluded from the male and female GO analyses to identify categories unique to each sex.

Adolescent binge ethanol led to the cryptic transcription of 30 genes in males and 26 in females (Figure 4A; Supplementary Table 9). Of these, 4 genes were cryptically transcribed in both sexes (*p* = 8.2e-9): *Ccdc124*, *Per1*, *Snap25*, and *Cplx1*. Interestingly, *Snap25* and *Cplx1* have been implicated in memory function (Hou et al., 2004; Söderqvist et al., 2010; Gao et al., 2015; Ramos-Miguel et al., 2017; Ying et al., 2017; Irfan et al., 2019; Wang et al., 2019; Gopaul et al., 2020), and play a role in brain development (Bark et al., 1995; Johansson et al., 2008; Salimi et al., 2008). In both males and females, *Snap25* also showed a significant decrease in gene expression due to binge ethanol treatment (Supplementary Table 1). *Per1* has also been previously shown to influence ethanol consumption (Gamsby et al., 2013) and was shown in a GWAS study to predict problematic alcohol use (Baranger et al., 2016).

The 26 genes that were uniquely cryptically transcribed in males, and the 22 genes that were uniquely cryptically transcribed in females were used as input for male-and female-specific gene ontology analysis. In males, gene categories that were over-represented due to binge ethanol treatment included calmodulin binding (molecular function), histone deacetylase binding (molecular function), and positive regulation of long-term potentiation (biological process; Figure 4B). Gene categories over-represented in females included transcription coactivator activity (molecular function) and regulation of release of sequestered calcium into cytosol (biological process; Figure 4C). Full gene ontology results for males and females can be found in Supplementary Table 10.

### 2.6. Adolescent binge ethanol induces differential H3K36 trimethylation of genes related to calcium channel activity and action potential in males

**Figure 5:**
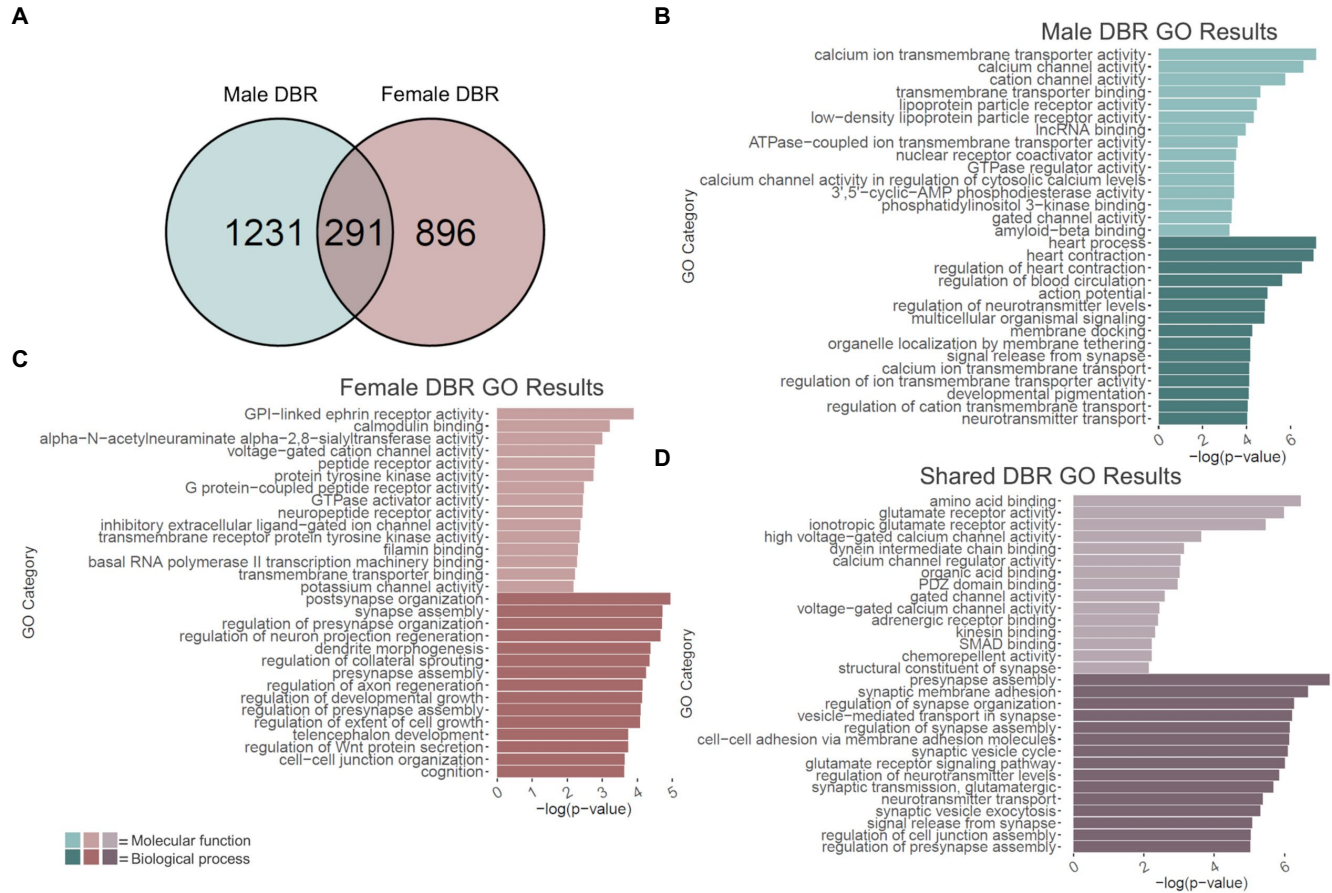
GO analysis of genes showing differentially bound H3K36me3 regions (DBR). **(A)** Number of genes showing differential binding of H3K36me3 due to adolescent binge ethanol in males and females at *p* < 0.05. Fisher’s exact test determined male and female gene overlap to be significant, *p* = 2.9e-131. **(B)** GO analysis of genes showing differential binding of H3K36me3 unique to males. **(C)** GO analysis of genes showing differential binding of H3K36me3 unique to females. **(D)** GO analysis of genes showing differential binding of H3K36me3 that are shared between males and females.

Changes in H3K36me3 due to adolescent binge ethanol were assessed with DiffBind. Using a cutoff of value of *p* < 0.05 to generate a gene list with a length sufficient for downstream analysis, 1,522 total genes were differentially bound in male PFC (Supplementary Table 11). 1,231 of these genes were unique to males (Figure 5A) and served as the input for male-specific gene ontology analysis. As with our RNA-seq dataset, the first 15 molecular function and biological process categories were selected to represent our ChIP-seq gene ontology results (Figure 5B). The full gene ontology results can be found in Supplementary Table 12. Molecular function categories of genes that were over-represented due to adolescent binge ethanol in males included calcium channel activity, long non-coding RNA binding, and nuclear receptor coactivator activity. Top biological process categories included action potential, regulation of neurotransmitter levels, and signal release from synapse. Although a Fisher’s Exact test showed the gene lists significantly overlapped, we were surprised to see only 27 genes were differentially expressed in our RNA-seq analysis and also showed altered H3K36me3 regulation (*p* = 5e-03, Table 1). Forty-nine genes that showed differential exon usage also showed altered H3K36me3 regulation (*p* = 7.5e-07, Table 1). Three genes that showed altered H3K36me3 regulation were cryptically transcribed in males (*Triqk*, *Dnajc21, Dclk3, p* = 0.16, Table 1). In males, *Penk, Lss, and Slc6a6* were the only genes showed differential trimethylation of H3K36, differential gene expression, and differential exon use (Table 1). No genes were shared across all four analyses.

### 2.7. Adolescent binge ethanol induces differential H3K36 trimethylation at calmodulin binding, synapse assembly, and cognition related genes in females

In females, DiffBind analysis showed that adolescent binge ethanol caused 1,187 total genes to be differentially associated with H3K36me3 in female PFC (Supplementary Table 11). Eight hundred ninety-six unique genes showed altered H3K36me3 associations in females, and these were used as input for female-specific gene ontology analysis (Figure 5A). Molecular function categories that were over-represented due to binge ethanol treatment included calmodulin binding, GPI-linked ephrin receptor activity, and inhibitory extracellular ligand-gated ion channel activity (Figure 5C). Over-represented biological process categories included synapse assembly, dendrite morphogenesis, and cognition (Figure 5C). Notably, adolescent binge ethanol impacted 20 genes associated with calmodulin binding and 30 genes associated with cognition (Figure 5C and Supplementary Table 13). Calmodulin plays a critical role in long-term potentiation and memory function (Ataei et al., 2015), and thus, disruption to the regulation of these genes could contribute to ethanol’s impact on memory. Similar to our male comparison, in females only 17 genes that were differentially expressed in our RNA-seq analysis showed altered H3K36me3 (*p* = 0.2, Table 1). Seventy-five genes that showed differential exon usage showed altered H3K36me3 (*p* = 5.5e-14, Table 1), and only *Cplx1* was differentially trimethylated at H3K36 and cryptically transcribed (*p* = 0.61, Table 1). In female ethanol-treated animals, four genes were found significant in our differential H3K36me3 analysis, differential gene expression analysis, and differential exon use analysis: *Smarca2, Hsph1, Unc13a, and Ipo9* (Table 1). No genes were shared across all four analyses.

### 2.8. Adolescent binge ethanol induces dysregulation of H3K36me3 at synaptic-related genes in both males and females

Of the 1,522 genes that showed altered H3K36me3 in males and 1,187 genes that showed altered H3K36me3 in females, 291 of those genes were shared between both sexes (*p* = 2.9e-131, Figure 5A). Gene ontology analysis of these 291 genes indicated that they overwhelmingly played a role in synaptic transmission and membrane excitability (Figure 5D). Molecular function categories included glutamate receptor activity, voltage-gated calcium channel activity, and structural constituent of synapse. Biological process categories included presynapse assembly, synaptic membrane adhesion, glutamate receptor signaling pathway, and signal release from synapse. While not within the top 15 biological function categories, adolescent binge ethanol also induced changes to H3K36 trimethylation in males and females at 16 genes related to learning or memory (GO:0007611): *Phb2, Adcy1, Ptgs2, Adcy8, Nrxn3, Nrxn1, Plcb1, Mme, Cacna1c, Amfr, Dcdc2, Gria1, Grm7, Brsk1, Ep300, Htt* and eight genes related to calmodulin binding (GO:0005516): *Adcy1*, *Adcy8*, *Plcb1*, *Cacna1a*, *Cacna1c*, *Ubr4*, *Grm7*, *Invs* (Supplementary Table 14). Interestingly, both categories were also significantly over-represented in the separate male and female analyses, although each sex saw differential H3K36me3 antibody binding at different genes (Supplementary Tables 12, 13). This gene-level functional analysis shows that adolescent binge ethanol produces a striking response of H3K36me3 dysregulation at genes critical for synaptic function. Given the role of H3K36me3 in memory-related processes described above (Butler and Dent, 2012; Sessa et al., 2019), and the ethanol-induced changes in H3K36me3 regulation we see in genes relating to membrane excitability, synaptic activity, and memory, these results provide evidence that link adolescent binge ethanol to H3K36me3 changes, and may link H3K36me3 changes to adolescent binge ethanol-induced memory deficits.

## 3. Discussion

Epigenetic regulation of gene expression provides an attractive mechanism for driving long-term behavioral and cognitive changes following binge ethanol. Epigenetic modifications can positively or negatively impact gene transcription, and are dynamic marks that are affected by environmental stimuli, such as stress or alcohol and illicit drug use (Jaenisch and Bird, 2003; Liu et al., 2008; Karlić et al., 2010; Hitchcock and Lattal, 2014). By altering transcription, epigenetic alterations provide a mechanism for regulating downstream gene expression, which in turn affects behavioral outcomes (Lester et al., 2011). To our knowledge, the studies here are the first to assess concurrent changes to gene expression and epigenetic regulation in the PFC of binge ethanol exposed adolescents. We assessed differential gene expression and ethanol-induced changes to H3K36me3, an epigenetic mark associated with memory function, alternative splicing, and cryptic transcription. These analyses were all carried out within the same tissue samples, to get a within-animal comparison of ethanol effects at both the chromatin and mRNA regulation levels. Our work shows that adolescent binge ethanol altered the regulation and expression of genes important for synaptic function.

Males and females both showed ethanol-induced changes to H3K36me3 at genes related to synaptic transmission and membrane excitability. Dysregulation of these genes could impede cell signaling pathways or synaptic pruning processes required for ongoing PFC development throughout adolescence. Previous studies have shown accelerated gray matter loss in the frontal cortex after adolescent alcohol exposure, indicating that synaptic pruning may be disrupted by binge levels of ethanol (Luciana et al., 2013; Squeglia et al., 2014; Pfefferbaum et al., 2018; El Marroun et al., 2021). A prior report showed that during a cognitive task, adolescents who binge drank showed differential brain region utilization despite similar performance, which may reflect the need to recruit more neural activity for successful performance (Squeglia et al., 2011). Males and females showed different responses, with male binge drinkers showing greater BOLD responses than controls, and female binge drinkers showing decreased BOLD responses compared to controls within the bilateral frontal, anterior cingulate, temporal, and cerebellar cortices (Squeglia et al., 2011). Altered regulation of genes involved in synaptic transmission and membrane excitability could potentially explain these ethanol-induced neuroimaging and behavioral changes. Notably, females showed much smaller fold changes in the genes where H3K36me3 was altered by ethanol compared to males, supporting our previous ELISA data where only ethanol-treated males showed global H3K36 decreases in the PFC (Wolstenholme et al., 2017).

Additionally, both sexes also showed decreased gene expression and cryptic transcription of *Snap25* with binge ethanol exposure*. Snap25* is part of the SNARE complex, and aids in calcium-induced release of neurotransmitters into the synaptic cleft (Najera et al., 2019). It has been associated with psychiatric outcomes such as ADHD and schizophrenia, both characterized in part by deficits in working memory (Najera et al., 2019; Kofler et al., 2020). In a post-mortem study, AUD patients showed lower protein levels of the Snap25b isoform. *Snap25* switches in expression from the *Snap-25a* isoform in early life to the *Snap-25b* isoform in adulthood (Bark et al., 1995), and this switch is influenced by sex (Irfan et al., 2019). Compared to males, females showed increased protein levels of the SNAP-25a isoform remaining at 4 weeks of age in the hippocampus (Irfan et al., 2019). *Snap25* deficiency studies have shown that the transition to *Snap-25b* is crucial for proper neurodevelopment – elimination of *Snap-25b* expression leads to impaired short-term plasticity (Johansson et al., 2008), and reduced magnitude of LTP (Irfan et al., 2019; Gopaul et al., 2020). The ethanol-induced altered expression of *Snap25* could directly induce the long-term working memory deficits seen after adolescent binge ethanol exposure. Not only could a change in the shift of *Snap25* isoforms induce changes in brain development and structure, but a polymorphism in *Snap25* has also been directly implicated in reduced gray matter of the cingulate cortex (Söderqvist et al., 2010) and reduced and working memory function in children and adolescents (Söderqvist et al., 2010; Gao et al., 2015).

*Cplx1* also showed evidence of cryptic transcription in males and females after exposure to adolescent binge ethanol. Complexins are SNARE-binding proteins and modulate calcium-induced vesicle release (McMahon et al., 1995; Reim et al., 2001; Chen et al., 2002). *Cplx1* is crucial for neurodevelopment, and similar to *Snap25*, also displays an age-dependent shift in expression. A study in the dorsolateral PFC showed that *Cplx1* increased in expression from 0 to 25 years – associated with an increase in synaptic maturation (Salimi et al., 2008). Interestingly, gene expression of complexins were found to be decreased in the PFC after prenatal (Barr et al., 2005) and perinatal (Zink et al., 2009) ethanol exposure. Given its role in both neurotransmitter release and PFC development, ethanol-induced changes to *Cplx1* could also contribute to ethanol’s impacts on memory.

H3K36me3 plays a role in memory consolidation within the PFC (Gräff et al., 2012). H3K36me3 is subjected to modulation by experience, as its level increased in the PFC after testing mice for recent (24 h) and remote (7 day) memory with a NOR task (Gräff et al., 2012). When histone modifications were pharmacologically blocked, memory consolidation was impaired, and this impairment was associated with a decrease in H3K36me3 levels (Gräff et al., 2012). Additionally, increases in global H3K36me3 levels were seen in transgenic animals with increased memory function, and when training time prior to NOR was increased, suggesting the level of H3K36me3 may determine how well a memory is consolidated (Gräff et al., 2012). Furthermore, a separate study showed that when H3K36me3 levels were reduced in a mouse model, deficits in NOR and decreased expression of memory-related genes occurred (Sessa et al., 2019). Ultimately, these data presented here suggest that adolescent binge ethanol perturbs H3K36me3’s regulation of genes important for synaptic function and memory processes and/or induces cryptic transcription of memory-related genes *Snap25* and *Cplx1*. These molecular changes could potentially be the mechanism by which ethanol induces persistent memory deficits.

Notably, a number of synaptic and memory-related gene ontology categories were repeated in the male, female, and the shared differential H3K36me3 analyses – although not all categories were within the top 15 for each analysis, calmodulin binding (GO:0005516), dendrite morphogenesis (GO:0048813), glutamatergic synaptic transmission (GO:0035249), negative regulation of synaptic transmission (GO:0050805), axon guidance (GO:0007411), learning or memory (GO:0007611) and cognition (GO:0050890) were seen significantly over-represented in each analysis (Supplementary Tables 12–14). This pattern was also seen in our RNA-seq differential genes expression analyses. For example, males and females showed different genes that were differentially expressed, but were all contained within the extracellular matrix structural constituent GO category (GO:0005201, Supplementary Tables 2–3). However, four of the same genes (*Kdr*, *Eng*, *Adamts6*, and *P4ha1*) in the extracellular matrix category (GO:0030198) were differentially expressed in both males and females (Supplementary Table 4). This may suggest that ethanol impacts the same pathways in males and females, but may do so through different mechanisms, or could suggest that ethanol influences a number of genes related to a pathway in both sexes, but these genes influence the expression of other downstream genes in a sex-specific manner. One study suggests that transcription factors have sex-biased regulatory targets (Lopes-Ramos et al., 2020) – because ethanol can alter transcription factor activity (Miles, 1995), it is possible that ethanol-induced changes to transcription factors may influence different genes in males and females. Another study saw sexual dimorphism in glutamatergic signaling-related genes within the nucleus accumbens after binge ethanol exposure, indicating the transcriptional response to ethanol is influenced by sex (Finn et al., 2018). Alternatively, our results may reflect that the same altered processes in males and females are altered, but due to stochastic variation in gene expression and possible low sample numbers, different genes, but the same biological pathways or molecular functions are identified between the sexes. While many RNA-sequencing studies have supported our results in finding sex-specific effects and/or little overlap between male and female gene lists (Labonté et al., 2017; Finn et al., 2018; Hitzemann et al., 2020), many other studies only include male subjects (Mulligan et al., 2017; Erickson et al., 2019; Brenner et al., 2020; Farris et al., 2020; Krishnan et al., 2022), and more studies including females will be required to parse out ethanol’s sex-dependent influence on gene expression.

Both males and females showed ethanol-induced changes to genes involved in the extracellular matrix. The extracellular matrix regulates cellular migration and axonal growth, and stabilizes myelinated tracts (Zimmermann and Dours-Zimmermann, 2008) – thus underlying its role in adolescent PFC development characterized by synaptic pruning and increased myelination (Spear, 2000). During early and adolescent brain development, the extracellular matrix supports neurogenesis, cell migration, axonal growth, and synaptogenesis, but in adulthood inhibits these reorganization processes (Gundelfinger et al., 2010). This restriction in adult plasticity is induced by the development of perineural nets, and corresponds with the end of the critical period where neuronal circuits are shaped by experience (Fawcett, 2009). Given its role in brain development and plasticity, alterations in the structure and function of the extracellular matrix could lead to lasting changes in the brain and have been suggested to underlie mental health, neuropsychiatric, or neurodegenerative disease (Lubbers et al., 2014). Previous studies have shown ethanol’s negative impact on the extracellular matrix in both adolescents (Coleman et al., 2014; Nato et al., 2014) and adults (Hitzemann et al., 2020), with extracellular matrix-related genes being identified in a number of AUD or substance use disorder GWAS studies (Adkins et al., 2017; Peng et al., 2021). Our study helps to support ethanol’s impact to the extracellular matrix.

Males and females both showed differential expression of a number of genes relating to the unfolded protein response (Figure 2D). Previous studies have shown that decreased levels of H3K36me3 allow for the production of cryptic transcripts (Carrozza et al., 2005; Butler and Dent, 2012; Carvalho et al., 2013), which associate with ribosomes (Wei et al., 2019), and likely become misfolded proteins due to their truncated nature. Therefore, this ethanol-induced response may be related to increased levels of cryptic transcription occurring.

We chose to do a differential exon use analysis using DEX-seq, due to H3K36me3’s proposed role in alternative splicing (Zhou et al., 2014; Xu et al., 2021). Here, we saw that genes showing evidence of differential exon use were involved in gene ontology categories related to tubulin binding and dendrite development in both males and females. When comparing the gene lists across analyses, we saw the most overlap in genes that showed differential exon use due to binge ethanol and genes where H3K36me3 was impacted by ethanol. In males, 49 genes showed ethanol-induced changes to H3K36me3 and differential exon use (*p* = 7.5e-07), and in females, 75 genes showed this overlap (*p* = 5.5e-14). This overlap could potentially be due to H3K36me3’s proposed role in alternative splicing (Zhou et al., 2014; Xu et al., 2021). We do, however, caution that given our sequencing protocol (poly-A selected, 150 bp reads), there may be some level of bias in alignments given our relatively short 150 bp read length. The impact of adolescent binge drinking on alternative splicing, and whether this is mediated through a H3K36me3 mechanism will need to be more thoroughly investigated in the future, perhaps with specific genes that showed both ethanol-induced differential exon use and altered H3K36me3 regulation or with long read RNA sequencing.

Given the critical role H3K36me3 plays in regulating cryptic transcription, we initially hypothesized that genes showing evidence of cryptic transcription would largely overlap with the same genes where we saw ethanol-induced differential binding of H3K36me3. However, there was little overlap of gene loci where the H3K36me3 antibody was differentially bound and genes that were cryptically transcribed – in males, only 3 genes that showed ethanol-induced changes to H3K36me3 were cryptically transcribed (*p* = 0.16), and in females, only one gene was found in both analyses (*p* = 0.61, Table 1). Similarly, there were less than 30 genes that were both differentially expressed and differentially bound by H3K36me3 in males (*p* = 5e-03) or females (*p* = 0.2, Table 1). H3K36me3 is deposited co-transcriptionally, and unlike most other histone marks, it does not strictly activate or repress gene transcription due to its presence at the gene promotor (DiFiore et al., 2020). The influence of H3K36me3 on gene expression levels has yet to be fully elucidated – gene-body marking by H3K36me3 does not always correlate with gene expression levels (Pu et al., 2015), and in one study, most DEGs did not exhibit obvious changes in H3K36me3 levels (Zhang et al., 2021). Some have suggested that decreases in H3K36me3 could leave the chromatin primed for greater flexibility in chromatin accessibility (Simon et al., 2014; Pu et al., 2015; Zhang et al., 2021). In two studies, high gene-body levels of H3K36me3 have been associated with transcriptional stability and few changes in gene expression, whereas low gene-body levels of H3K36me3 were associated with more gene expression changes (Pu et al., 2015; Zhang et al., 2021). It is possible that ethanol did not disrupt H3K36me3 gene-body levels to the point of eliciting strong impacts to gene expression levels, and thus we did not see a large overlap of genes where levels of H3K36me3 were altered and genes that were differentially expressed due to binge ethanol. However, suggested by the studies above, decreases in H3K36me3 gene-body levels could still lead to altered chromatin regulation.

A study using a similar approach to generate ChIP-seq and RNA-seq data from within the same tissue also suggested that effects at the chromatin regulation level do not always accurately predict levels of gene expression (Reshetnikov et al., 2021). This could potentially be explained by the differences in H3K36me3 half-life versus the half-life of mRNA. Zheng et al. (2014) estimated the half-life of H3K36me3 in HeLa cells at about 57 h. The half-life of mRNA varies based on a variety of factors, but is estimated to last up until 10 h in mammalian cells (Yang et al., 2003; Chen et al., 2008; Sharova et al., 2009; Tani et al., 2012). This could suggest H3K36me3-regulated genes might not accurately represent the full mRNA profile, i.e., H3K36me3 may remain on the chromatin long after the associated mRNA has been degraded. Thus, H3K36me3 may provide a “molecular memory” of previous transcriptional events, similar to those seen with H3K4 methylation in Ng et al. (2003). Another study that assessed chromatin and gene expression changes within the same PFC tissue saw that H3K4me3 showed low correlation with gene expression, but that correlation improved when looking at specific cell types (Reshetnikov et al., 2020, 2021). Thus, an alternate explanation for the small overlap between genes where H3K36me3 levels were altered and differentially expressed genes could be that the data was obscured by looking at all cells comprising the PFC instead of cell-specific populations.

## 4. Materials and methods

### 4.1. Animals

Male and female DBA/2J mice (*n* = 66) from Jackson Laboratory arrived in the Virginia Commonwealth University vivarium at postnatal day 19–21 (Bar Harbor, ME, United States). DBA/2J mice were used as they show a more robust acute response to binge ethanol than the more commonly used C57BL/6J strain – DBA/2J mice show increased sensitivity to ethanol in a loss of righting reflex test (Linsenbardt et al., 2009), as well as increased locomotor activity compared to C57/B6 mice (Phillips et al., 1994; Kerns et al., 2005). Importantly, this strain also shows a more prominent decrease in myelin gene expression (Kerns et al., 2005; Goudriaan et al., 2020) – important to our model as myelin decreases have been widely shown in human studies of adolescent binge drinking (De Bellis et al., 2005; Medina et al., 2008; Pfefferbaum et al., 2018; El Marroun et al., 2021). Mice were housed 4/cage in same sex cages in an AALAC-accredited facility under 12-h light/dark cycles with food and water available *ad libitum* for the entire experiment. After a week acclimation to the animal facility, mice were habituated to the gavage procedure with 0.1% saccharin on PND 27 and 28 and then divided into two treatment groups: ethanol treated and control. Mice were dosed with 4 g/kg ethanol (25% w/v in water by gavage) or water intermittently (2 days on/2 days off) on PND 29, 30, 33, 34, 37, 38, 41, and 42. PFC tissue was collected 24 h after the last dose of ethanol (3–6 h into the animals’ light cycle). A triangle-shaped wedge of tissue above the corpus callosum was collected anterior to Bregma 0.5 mm to represent the PFC, consistent with typical PFC dissection regions for the Wolstenholme lab (Wolstenholme et al., 2011, 2017). This wedge contains prelimbic, infralimbic and medial PFC as well as anterior cingulate. Tissue was collected 24 h after the last dose based on a previous study in our lab, where we saw significant decreases in global H3K36 and H3K36-related gene expression at this timepoint (Wolstenholme et al., 2017). Collecting tissue at this timepoint allowed us to investigate the immediate impacts of adolescent binge ethanol use to see whether molecular changes during a critical developmental period could inform the long-term cognitive deficits seen in adulthood. At this dose, blood ethanol concentrations reached 313 mg/dL 1 h after gavage (Wolstenholme et al., 2017). All animal housing and care was conducted with the approval of the Virginia Commonwealth University IACUC Committee and in accordance with the NIH Guide for the Care and Use of Laboratory Animals (National Research Council, 2011).

For our ChIP-seq and RNA-seq studies, 66 total animals were used. To ensure enough DNA input for high quality sequencing data, we combined three PFCs from each treatment group and sex into a single sample. Each combined tissue sample was homogenized and divided in half between ChIP-seq and RNA-seq sample preps so that we were able to link chromatin-level changes with gene expression-level changes from within the same tissue. Five biological replicates (of three pooled PFCs) were obtained for water-treated male and female groups, and six biological replicates (of three pooled PFCs) were obtained for ethanol-treated males and females, resulting in *n* = 22 pooled samples for each of our sequencing studies (Figure 1).

Sex-specific responses to ethanol have been well-noted (Squeglia et al., 2011; Labonté et al., 2017; Wolstenholme et al., 2017; Finn et al., 2018; Tavares et al., 2019; Flores-Bonilla and Richardson, 2020; Hitzemann et al., 2020; Radke et al., 2021; Bent et al., 2022), and because of this, we had an *a priori* hypothesis that ethanol induces different molecular changes in males and females. Thus, our male and female analyses were run separately. These sex-specific responses are exemplified with previous ELISA data from our lab showing that global H3K36 methylation levels were significantly decreased after adolescent binge ethanol treatment in males only, and that males had more robust decreases in myelin-related gene expression (Wolstenholme et al., 2017). These sex-specific responses have been seen specifically in other RNA-seq studies, where males and females show little overlap in differentially expressed genes (Labonté et al., 2017; Finn et al., 2018). Another recent ethanol-associated RNA-seq study also chose to analyze the sexes separately, given the sex-specific effects in alcohol use (Hitzemann et al., 2020).

### 4.2. RNA isolation

Homogenized and divided tissue was immediately pelleted and flash frozen on liquid nitrogen. Total RNA was isolated from PFC tissue using STAT60 Reagent (Tel-Test, Friendswood, TX) and miRNeasy mini kit (Qiagen, Valencia, CA) according to the manufacturer’s protocol. RNA was quantified by measuring absorbance at 260 nm using a NanoDrop, and RNA integrity was assessed by electrophoresis using an Agilent 2100 Bioanalyzer (Agilent, Santa Clara, CA). All samples obtained an RNA Integrity Number (RIN) between 8.4 and 9.3.

### 4.3. RNA-seq library preparation and sequencing

RNA-seq data have been deposited with the Gene Expression Omnibus resource (Accession #GSE220746). Isolated RNA of pooled samples was sequenced by Novogene. To avoid non-biological experimental variation that could arise from sample batches, similar group representation was ensured prior to each processing stage (RNA isolation, library preparation, and lane assignment). Preparation of polyA-enriched cDNA libraries was conducted following standard protocols using NEBNext Ultra^™^ II Directional RNA Library Prep Kit (Illumina). Libraries were sequenced on an Illumina NovaSeq 6000 (2 × 150 bp paired-end reads). A summary of RNA-seq metrics can be found in Supplementary Table 15.

### 4.4. RNA-seq alignment and differential gene expression (DEG) analysis

Quality of FASTQ formatted samples files were assessed with FastQC (Andrews, 2010), and aligned using STAR v2.7.8a (Dobin et al., 2013) with GRCm38/mm10 reference genome. Annotations were obtained from ENSEMBL. Only reads that mapped to a single location were used in subsequent analysis. Raw read counts were compiled using featureCounts within the Bioconductor package Subread v2.0.2 (Liao et al., 2014). A pre-filtering step was applied to eliminate very low expressing genes prior to differential gene expression analysis, where at least two samples were required to have >10 counts. Normalization and differential expression analysis were carried out with DESeq2 v1.32.0 (Love et al., 2014). DESeq2 internally normalizes read counts using a median of ratios method (Love et al., 2014). Genes with a value of *p* < 0.01 were considered significantly altered and used in downstream bioinformatic analysis.

### 4.5. Differential exon usage

GFF annotation file containing collapsed exon counting bins was prepared from the ENSEMBL GRCm38/mm10 GTF file using the DEXSeq v1.38.0 (Anders et al., 2012) Python script dexseq_prepare_annotation.py with gene aggregation disabled. The number of reads overlapping each exon bin was then counted using the DEXSeq Python script dexseq_count.py, the GFF file, and each sample’s SAM file. Differential exon usage (DEU) analysis was then carried to compare ethanol-treated animals to water-treated animals using the DEXSeq R package standard analysis workflow. Genes with transcripts possessing at least one differentially utilized exon bin with a value of *p* < 0.001 were considered to be significantly altered and were used in downstream bioinformatic analysis.

### 4.6. Cryptic transcription analysis

To identify genes that displayed evidence of cryptic transcription in males and females, we followed the protocol outlined in Carvalho et al. (2013). Exon read counts generated with DEXSeq (Anders et al., 2012) were used to compare ethanol-treated and control animals for each sex. Briefly, we first identified all transcripts with significantly higher read counts for at least one exon in the EtOH treated group. Second, transcripts with significantly higher read counts for the first annotated and expressed exon (likely corresponding to upregulated full-length mRNAs) were discarded. Third, the new cryptic TSS was defined as the first significantly higher exon, and all transcripts that showed significantly higher read counts for <60% of the downstream exons were filtered out. In addition to Carvalho’s parameters, we required the first differentially used exon to be among the first half of all exons. The resulting gene lists of cryptically transcribed genes were used for downstream bioinformatics analyses.

### 4.7. Chromatin immunoprecipitation

Prior to ChIP, the H3K36me3 antibody (Active Motif #61101, Carlsbad, CA) was validated using qPCR to verify H3K36me3 presence or absence at specific genomic locations against IgG (Supplementary Figure 1A). The same antibody lot number was used for all ChIP reactions. H3K36me3-ChIP was performed on PFC tissue using the MAGNA ChIP Hi-Sens kit (Millipore, Burlington, MA), following the manufacturer’s instructions. Briefly, tissue from 3 combined PFCs was dounce homogenized on ice, divided in half, and pelleted. One half of each sample was flash frozen and reserved for RNA-seq. The other half of each sample was subjected to fixation, where DNA was cross-linked to histones by incubating cells in a 1% formaldehyde solution at 37°C for 10 min. The formaldehyde reaction was quenched using glycine, and tissue was washed 3x with PBS containing protease inhibitors (Millipore, Burlington, MA). Cell lysis buffer was added to isolate nuclei. Prepared chromatin was sonicated using a Bioruptor Pico (Diagenode, Denville, NJ) for 12 cycles (30 s on/30 s off). Fragment size was verified on 2% agarose gel to be between 200 and 1,000 bp. After sonication, samples were stored at −20°C to await immunoprecipitation, and a portion of sonicated chromatin from each sample was reserved as an input reference. For immunoprecipitation, sonicated chromatin was incubated overnight with 2 μg of H3K36me3 antibody (Active Motif, Carlsbad, CA) bound to Protein A/G magnetic beads with end-over-end rotation. After incubation, DNA was eluted from beads in elution buffer with proteinase K. DNA was extracted using phenol:chloroform:isoamyl alcohol (Thermo Fisher, Waltham, MA) and purified with ethanol precipitation. Pellets were resuspended in 20 μL TE buffer and stored at −20°C. To obtain sufficient DNA for sequencing, 8 technical replicates from each sonicated sample were subjected to immunoprecipitation. DNA from all 8 technical replicates were combined and concentrated using a speed-vacuum prior to library preparation. Independent control samples were run alongside each set of ChIP reactions to ensure successful chromatin immunoprecipitation.

### 4.8. ChIP-seq library preparation and sequencing

ChIP-seq data have been deposited with the Gene Expression Omnibus resource (Accession #GSE220746). To avoid non-biological experimental variation that could arise from sample batches, similar group representation was ensured prior to each processing stage (sonication, ChIP, library preparation, and lane assignment). Input and H3K36me3-enriched samples were sequenced by the VCU Genomics Core Facility for library preparation and sequencing. Preparation of libraries were conducted following standard protocols using the Accel-NGS 2S PLUS DNA Library kit (Swift). Samples were sequenced on a NextSeq 2000 (Illumina) using 150 base pair single-end reads at greater than 50 million reads. A summary of ChIP-seq metrics can be found in Supplementary Table 15.

### 4.9. ChIP-seq alignment and differential binding analysis

Quality of FASTQ formatted samples files were assessed with FastQC. Adapter sequences and reads with quality scores <20 were removed using CutAdapt (Martin, 2011) and aligned using Bowtie2v2.4.1 (Langmead and Salzberg, 2012) with GRCm38/mm10 reference genome. To prevent inaccurate representation of repetitive genomic regions, reads that aligned to blacklisted regions were discarded, as defined by ENCODE to be a set of regions in the genome that have anomalous, unstructured, or high signal in next-generation sequencing experiments independent of cell line or experiment (Amemiya et al., 2019). Only reads that mapped to a single location were used in subsequent analysis. Peaks were identified using SICERv1.1 (W200, G600) (Xu et al., 2014). As with our RNA-seq analysis, male and female analyses were run separately. Normalization and differential peak binding analysis were carried out using the Bioconductor package DiffBind v3.4.11, and annotations were obtained using the Bioconductor package ChIPSeekerv1.28.3 (Yu et al., 2015). Genes with a value of *p* < 0.05 were considered significantly altered and used in downstream bioinformatic analysis to expand our gene list for hypothesis-generating analyses. Representative samples of H3K36me3 read coverage are shown in Supplementary Figure 1B.

### 4.10. Bioinformatics analysis

Downstream bioinformatics analysis was carried out similarly for gene lists derived from differential expression, differential exon use, cryptic transcription, and differential binding analyses. Gene ontology over-representation analysis was determined using ToppFun (Chen et al., 2009). Gene sets were filtered based on the number of genes within each category (min = 4, max = 500) and value of *p* of <0.05. Lists were further filtered by gene list hits. For analyses with ≥100 genes, categories containing ≥3 hits were included and the top 15 molecular function and biological process categories are represented in our figures. For analyses with ≤100 genes, categories containing ≥2 hits were included, and the top 5 molecular function and biological process categories are represented. Categories that had identical query gene lists and similar category names were removed to reduce repetitiveness. Full gene ontology tables can be found in Supplementary Tables 2–4, 6–8, 10, 12–14. Within each analysis, male and female gene lists were compared using a Fisher’s exact test to determine whether there was significant gene overlap of the two lists in comparison to the total number of genes in the mm10 genome. This same test was used to determine if there was significant overlap between each analysis within each sex. The Bioconductor package GeneOverlap v1.32.0 was used for these overlap calculations.

## 5. Conclusion

Overall, this study unveiled potential genes that may be contributing to adolescent binge ethanol-related memory deficits. As H3K36me3 plays an important role in memory, and was previously found to be decreased in the PFC after adolescent binge ethanol (Wolstenholme et al., 2017), we assessed ethanol-induced changes to this histone mark in the developing adolescent PFC. We further assessed ethanol-induced changes at the transcription level with differential gene expression and differential exon-use analyses, as well as cryptic transcription analysis – a marker of decreased H3K36me3. Importantly, all analyses were completed within the same tissue to view changes at both the epigenetic and gene expression levels.

**Figure 6:**
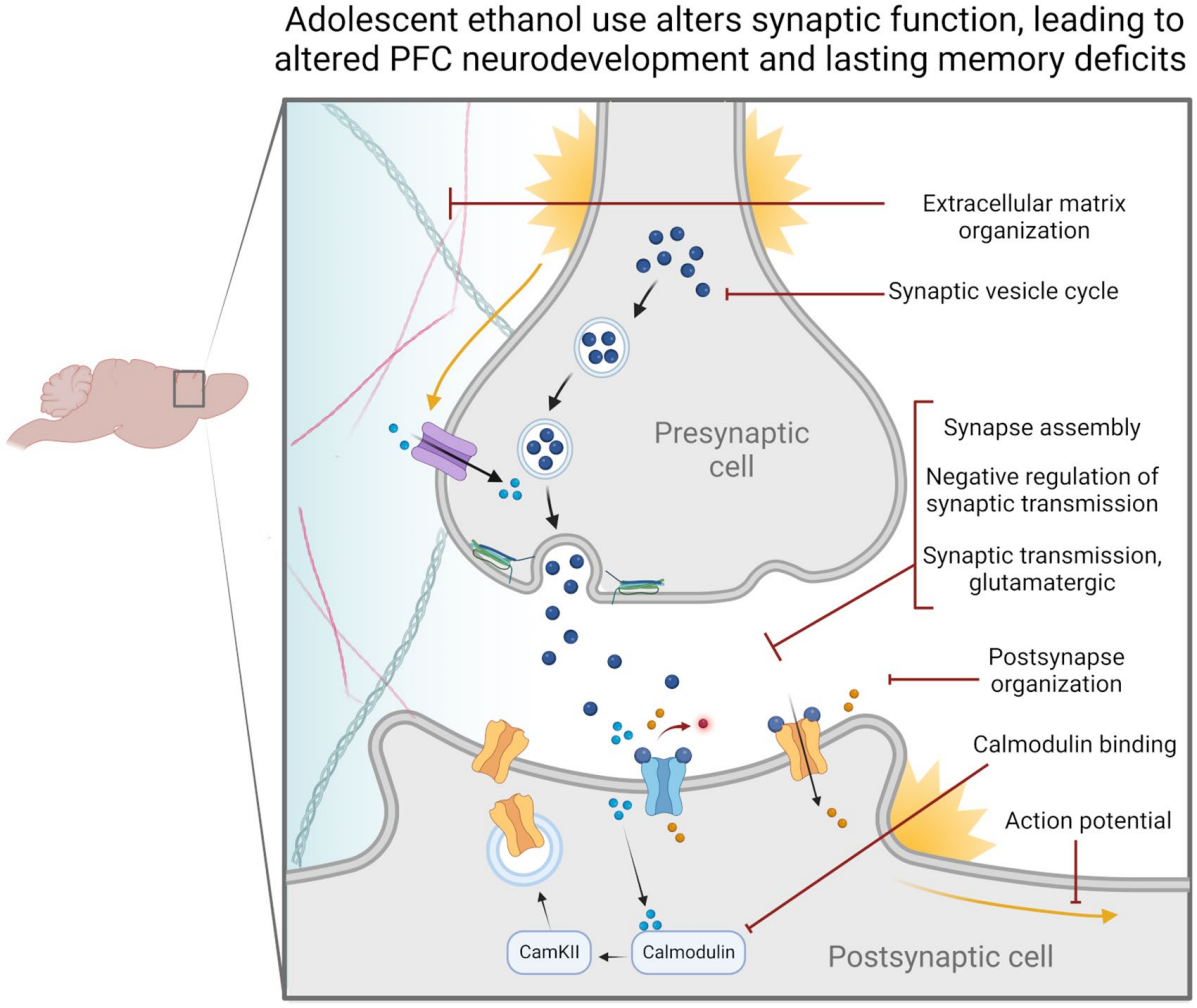
Adolescent binge ethanol alters the regulation and expression of genes relating to synaptic function. Our differential gene expression, differential exon usage, cryptic transcription and differential H3K36me3 bound loci analyses identified a number of gene ontology categories related to synaptic structure and function. These changes are likely reflected in presynaptic and postsynaptic cells as well as within the extracellular matrix and may be why persistent cognitive deficits occur after adolescent binge ethanol exposure. Categories shown were significantly over-represented in at least one analysis in both males and females, *p* < 0.05. Extracellular matrix organization – GO:0030198, Synaptic vesicle cycle – GO:0099504, Synapse assembly – GO:0007416, Negative regulation of synaptic transmission – GO:0050805, Synaptic transmission, glutamatergic – GO:0035249, Postsynapse organization – GO:0099173, Calmodulin binding – GO:0005516, Action potential – GO:0001508.

Through this set of experiments, we found changes to synaptic-related genes in all our analyses. Notably, H3K36me3 was dysregulated at synaptic-related genes. Dysregulation of synaptic-related gene expression could impede cell signaling pathways required for ongoing PFC development throughout adolescence and provide an avenue for cognitive deficits to occur in adulthood (Figure 6). We also found that *Snap25* and *Cplx1* showed evidence of cryptic transcription and that the former showed decreased gene expression in both males and females. These genes play a strong role in memory function and brain development, and similarly, their specific dysregulation could underlie adolescent ethanol-induced cognitive phenotypes (Johansson et al., 2008; Salimi et al., 2008; Söderqvist et al., 2010; Gao et al., 2015; Irfan et al., 2019; Gopaul et al., 2020). Further experiments will be required to assess the exact role of these genes in ethanol-induced memory deficits, and whether they are implicated in other ethanol-related behaviors.

## Data availability statement

The datasets presented in this study have been deposited with the Gene Expression Omnibus resource (Accession #GSE220746). Full gene lists can be found in Supplementary material.

## Ethics statement

The animal study was reviewed and approved by the Virginia Commonwealth University IACUC Committee and in accordance with the NIH Guide for the Care and Use of Laboratory Animals.

## Author contributions

EB and JW conceived and designed the study. EB executed, analyzed, and wrote the study with revision and editing assistance from JW. JW provided resources, experimental interpretation, and critical review of the manuscript. All authors contributed to the article and approved the submitted version.

## Funding

This research was supported by the National Institutes of Health, National Institute on Alcohol Abuse and Alcoholism, USA 1F31AA029259-01A1, R01AA026347, and P50AA022537.

## Conflict of interest

The authors declare that the research was conducted in the absence of any commercial or financial relationships that could be construed as a potential conflict of interest.

## Publisher’s note

All claims expressed in this article are solely those of the authors and do not necessarily represent those of their affiliated organizations, or those of the publisher, the editors and the reviewers. Any product that may be evaluated in this article, or claim that may be made by its manufacturer, is not guaranteed or endorsed by the publisher.

## Supplementary material

SUPPLEMENTARY TABLE 1 Differential gene expression results for male and female PFC.

SUPPLEMENTARY TABLE 2 Gene ontology analysis for the 342 genes differentially expressed in the PFC after adolescent binge ethanol in males only, *p* < 0.05.

SUPPLEMENTARY TABLE 3 Gene ontology analysis for the 376 genes differentially expressed in the PFC after adolescent binge ethanol in females only, *p* < 0.05.

SUPPLEMENTARY TABLE 4 Gene ontology analysis for the 45 genes differentially expressed in the PFC after adolescent binge ethanol in both males and females, *p* < 0.05.

SUPPLEMENTARY TABLE 5 Differential exon use results in male and female PFC.

SUPPLEMENTARY TABLE 6 Gene ontology analysis for the 461 genes showing differential exon usage in the PFC after adolescent binge ethanol in males only, *p* < 0.05.

SUPPLEMENTARY TABLE 7 Gene ontology analysis for the 759 genes showing differential exon usage in the PFC after adolescent binge ethanol in females only, *p* < 0.05.

SUPPLEMENTARY TABLE 8 Gene ontology analysis for the 41 genes showing differential exon usage in the PFC after adolescent binge ethanol in both males and females, *p* < 0.05.

SUPPLEMENTARY TABLE 9 Cryptically transcribed genes in male and female PFC after adolescent binge ethanol, *p* < 0.05.

SUPPLEMENTARY TABLE 10 Gene ontology analysis for the 26 cryptically transcribed genes in the PFC after adolescent binge ethanol in males and the 23 cryptically transcribed genes in the PFC after adolescent binge ethanol in females, *p* < 0.05.

SUPPLEMENTARY TABLE 11 Differential trimethylation of H3K36 results in male and female PFC. In males, 1522 total genes showed H3K36me3 changes after adolescent binge ethanol treatment (*p* < 0.05); 182 of these genes showed significant changes in H3K36me3 at multiple sites. In females, 1187 total genes showed H3K36me3 changes (*p* < 0.05), 113 of these genes showed significant changes in H3K36me3 at multiple sites.

SUPPLEMENTARY TABLE 12 Gene ontology analysis for the 1231 unique genes that showed differential trimethylation of H3K36 in the PFC after adolescent binge ethanol in males only, *p* < 0.05.

SUPPLEMENTARY TABLE 13 Gene ontology analysis for the 896 unique genes that showed differential trimethylation of H3K36 in the PFC after adolescent binge ethanol in females only, *p* < 0.05.

SUPPLEMENTARY TABLE 14 Gene ontology analysis for the 291 genes that showed differential trimethylation of H3K36 in the PFC after adolescent binge ethanol in both males and females, *p* < 0.05.

SUPPLEMENTARY TABLE 15 Summary of sample and alignment information for RNA-seq and ChIP-seq data.

SUPPLEMENTARY FIGURE 1 H3K36me3 ChIP antibody validation and sequencing coverage. A. H3K36me3 antibody (Active Motif) was validated using positive (Aldoa) and negative (Chr12dsrt) primers to visualize regions of expected high and low binding, respectively, and was compared to IgG (Millipore) to identify enrichment over non-specific binding. Graph shows representative sample. Aldo1a F: AGGTGGATGCACCATCACATT, Aldo1a R: TGCATTAGAGATGCGGAGCA, Chr12dst F: CACTGACACCTGACCTCGAT, Chr12dsrt R: ATGCCACAGCCCAAGTATGG. B. Representative ChIP read coverage of three genes that showed ethanol-induced changes to H3K36me3: Cacna1b, Lrrk2, and Ryr2.
