# Supplementary figures and images for: Adolescent binge ethanol impacts H3K36me3 regulation of synaptic genes
Source: Front Mol Neurosci. 2023 Mar 3;16:1082104. doi: 10.3389/fnmol.2023.1082104 (PMC10020663; doi:10.3389/fnmol.2023.1082104)

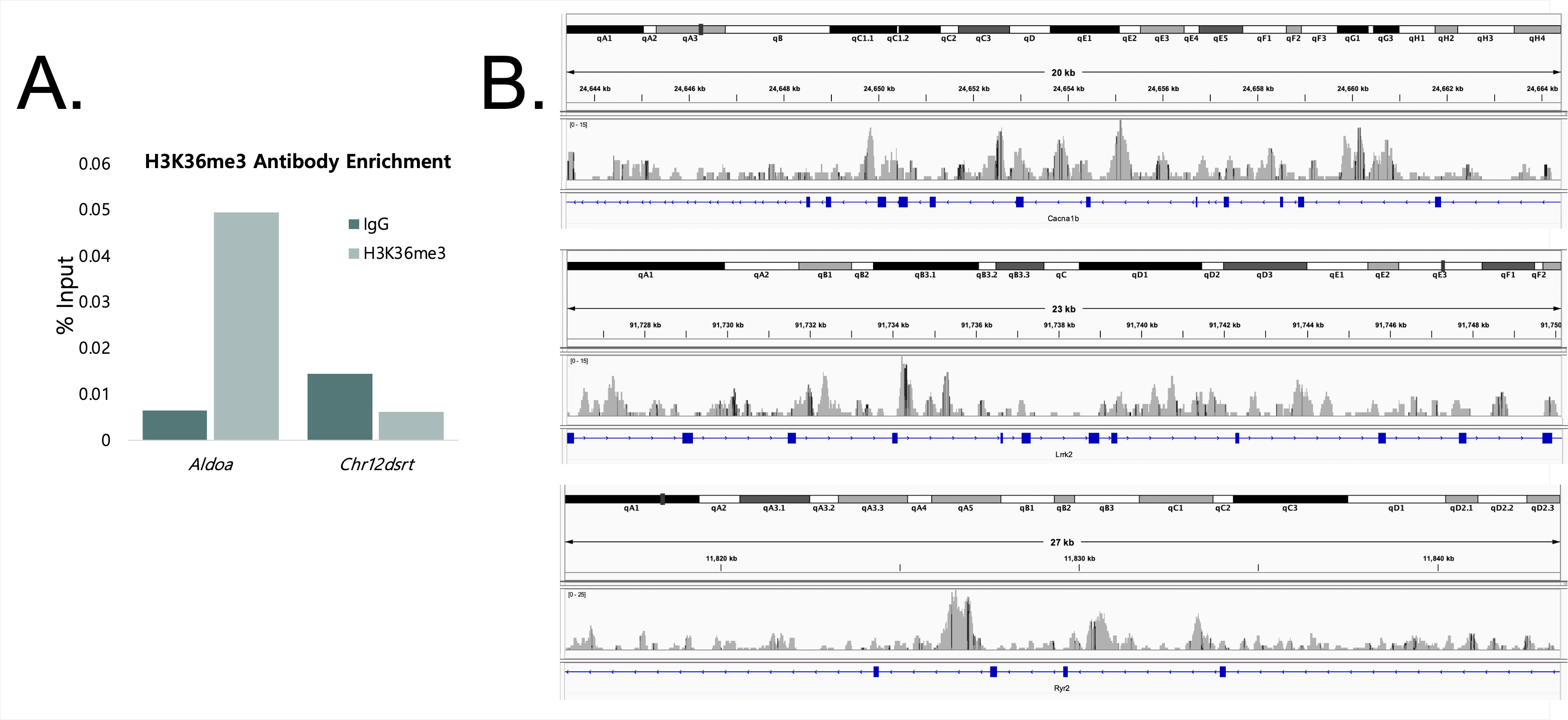

Supplement: SUPPLEMENTARY FIGURE 1 — H3K36me3 ChIP antibody validation and sequencing coverage. A. H3K36me3 antibody (Active Motif) was validated using positive (Aldoa) and negative (Chr12dsrt) primers to visualize regions of expected high and low binding, respectively, and was compared to IgG (Millipore) to identify enrichment over non-specific binding. Graph shows representative sample. Aldo1a F: AGGTGGATGCACCATCACATT, Aldo1a R: TGCATTAGAGATGCGGAGCA, Chr12dst F: CACTGACACCTGACCTCGAT, Chr12dsrt R: ATGCCACAGCCCAAGTATGG. B. Representative ChIP read coverage of three genes that showed ethanol-induced changes to H3K36me3: Cacna1b, Lrrk2, and Ryr2. [file Image_1.TIF]
